# Disrupted Endothelial Cell Layer and Exposed Extracellular Matrix Proteins Promote Capture of Late Outgrowth Endothelial Progenitor Cells

**DOI:** 10.1155/2016/1406304

**Published:** 2016-06-16

**Authors:** Jing Zhao, Claudia-Gabriela Mitrofan, Sarah L. Appleby, Nicholas W. Morrell, Andrew M. L. Lever

**Affiliations:** Department of Medicine, Addenbrooke's Hospital, University of Cambridge, Cambridge CB2 0QQ, UK

## Abstract

Late outgrowth endothelial progenitor cells (LO-EPC) possess a high proliferative potential, differentiate into vascular endothelial cells (EC), and form networks, suggesting they play a role in vascular repair. However, due to their scarcity in the circulation there is a requirement for* ex vivo* expansion before they could provide a practical cell therapy and it is currently unclear if they would home and engraft to an injury site. Using an* in vitro* flow system we studied LO-EPC under simulated injury conditions including EC activation, ischaemia, disrupted EC integrity, and exposed basement membrane. Perfused LO-EPC adhered to discontinuous EC paracellularly at junctional regions between adjacent cells under shear stress 0.7 dyn/cm^2^. The interaction was not adhesion molecule-dependent and not enhanced by EC activation. LO-EPC expressed high levels of the VE-Cadherin which may explain these findings. Ischaemia reperfusion injury decreased the interaction with LO-EPC due to cell retraction. LO-EPC interacted with exposed extracellular matrix (ECM) proteins, fibronectin and vitronectin. The interaction was mediated by integrins *α*5*β*3, *α*v*β*1, and *α*v*β*3. This study has demonstrated that an injured local environment presents sufficient adhesive signals to capture flow perfused LO-EPC* in vitro* and that LO-EPC have properties consistent with their potential role in vascular repair.

## 1. Introduction

Endothelial cells (EC) play an important role in regulating vascular homeostasis, modulating permeability, maintaining vascular tone, and responding to various stimuli by the production of bioactive substances [1]. Loss of endothelial integrity may cause a variety of deleterious consequences including acute events such as thrombus formation and predisposing to chronic pathology including transplant vasculopathy and atherosclerosis leading to complications such as coronary heart disease, stroke, and diabetes [2–5]. Endothelial integrity depends on a balance between the extent of endothelial cell injury and the capacity for endogenous repair. In healthy individuals, neighbouring mature endothelial cells can replicate locally and replace damaged cells [3]. However if injurious stimuli are prolonged and/or repeated or there is a large area of damage, endogenous repair may be inadequate [6] and require additional repair mechanisms.

Endothelial progenitor cells (EPC) could provide an alternative mechanism for maintenance and repair of damaged endothelium* in vivo*. Two types of EPC with distinct properties have been identified, early outgrowth EPC (EO-EPC) and late outgrowth EPC (LO-EPC) [7–11]. Early outgrowth EPC are short-lived cells (<2 weeks) and do not differentiate into EC* in vivo* but can restore endothelial function and enhance angiogenesis after tissue ischaemia via a paracrine effect [8, 12, 13]. However, they are a heterogeneous population of hematopoietic cells including monocyte-derived immune cells [12, 14, 15]; delivering large numbers of* ex vivo* expanded autologous EO-EPC might risk exacerbating immune response. LO-EPC, by contrast, are a homogeneous endothelial-like progenitor cell population that possess a high proliferative potential, differentiate into vascular endothelial cells, and form networks* in vitro* and* in vivo* [10, 16, 17]. We and others have shown that LO-EPC morphology and angiogenic function is preserved in patients with cardiovascular risk factors and patients with end stage renal failure [16, 18]. Their proliferation, differentiation, and tube forming ability are increased by laminar shear stress [19–22] suggesting that they may contribute to autologous vascular repair. However LO-EPC are not abundant in the circulation [7, 23]. To use them therapeutically LO-EPC would need to be expanded* ex vivo* to high concentrations before being delivered back into the circulation. The fate of LO-EPC after delivery including their ability to home to and engraft at a site of injury is not known.

Vascular damage is characterised by endothelial cell activation and dysfunction that may progress to detachment leading to loss of endothelial integrity [3, 24]. Endothelial cell damage markers including endothelial microparticles derived from activated or apoptotic cells and whole endothelial cells can be detected in the circulation [25]. Once the endothelial monolayer is disrupted, the basement membrane is exposed to blood flow. This layer provides the primary physical support for endothelial cells and is composed of collagen type IV, collage type I, fibronectin, vitronectin, laminin, and several proteoglycans (including heparin sulphate proteoglycan) [26]. These local changes may influence LO-EPC homing and engraftment. In this study, we investigated the dynamic interaction of LO-EPC with normal endothelial cells, activated endothelial cells or those undergoing simulated ischaemia reperfusion injury, and different extracellular matrix (ECM) proteins. Understanding the interaction of LO-EPC under simulated injury conditions* in vitro* and the mechanism of LO-EPC capture from flow will provide us with a critical view on the practicality of using LO-EPC for endogenous repair.

## 2. Materials and Methods

### 2.1. Cell Culture

This study had full ethical approval from the institutional review board of the Clinical School, University of Cambridge, and written informed consent was obtained from all volunteers. Late outgrowth EPC were isolated as previously described [16]. Briefly, mononuclear cells (MNC) were isolated from 40 mls venous peripheral blood by density-gradient centrifugation with Ficoll-paque-1.077 (GE Healthcare, UK). The mononuclear cells were plated in a culture flask coated with type I collagen (BD, UK) and cultured at 37°C under 5% CO_2_ atmosphere in endothelial basal medium (EBM) supplemented with SingleQuots (Lonza) and 20% Hyclone fetal calf serum (Fisher Scientific, UK). Nonadherent cells were removed after 3 days in culture and the medium was changed on alternate days. Colonies of LO-EPC appeared after 2 to 3 weeks in culture and exhibited typical cobblestone morphology. Once individual colony cell number reached 500–1000, the cells were passaged into a new collagen-coated flask. Subsequently cells were passaged at a 1 : 3 ratio into noncoated flasks. The medium was changed every other day. LO-EPC from passages 4–6 were used.

Human abdominal aorta endothelial cells (HAEC) were purchased from PromoCell, Germany. The cells were cultured in complete endothelial growth medium with 5% fetal calf serum (PromoCell). The medium was changed every other day. Cells from passages 3–6 were used.

### 2.2. Interaction of LO-EPC and HAEC under* In Vitro* Shear Flow

3 × 10^4^ HAEC were plated directly on Ibidi *μ*-Slide VI 0.4 Luer slides (Thistle Scientific LTD, UK) 48 hours before the experiments. HAEC were either left untreated, or treated with 0.05 ng/mL TNF*α* for 4 hours, or subjected to ischaemia for 4 hours followed by reperfusion overnight, before being connected to the flow system. The flow system was set up as previously described [27, 28]. Briefly, to perfuse the cells in the flow system, one end of the Ibidi slide was attached by silicon rubber tubing to an electronic valve, which allowed smooth switching between the LO-EPC suspension and wash buffer (1% BSA in DPBS, Sigma, UK) held in vertical syringe barrels, and the other end of the Ibidi slide was attached by silicon rubber tubing to a Harvard syringe pump (Harvard Apparatus, UK). The flow rate of 0.4 mL/min was pump-controlled. The equivalent shear stress (*τ*) exerted on Ibidi *μ*-Slide VI 0.4 Luer slide surface at a flow rate of 0.4 mL/min (Φ) was 0.7 dyn/cm^2^, which was calculated from the equation “*τ* = *η*176.1Φ”. *η* (dynamical viscosity) was 0.01 dyn·s/cm^2^. LO-EPC were labelled with Dil-Ac-LDL to distinguish them from HAEC after adhesion. After insertion of the Ibidi slide into the flow system, the slide was washed for 2 min with 1% BSA in DPBS (perfusion buffer). A total of 4 × 10^5^ labelled LO-EPC in 1.5 mL perfusion buffer were then perfused at a shear stress of 0.7 dyn/cm^2^ for 4 min. An additional 2 min wash was applied to remove nonadherent cells. The interaction of LO-EPC with endothelial cells was observed and recorded during the LO-EPC perfusion, and the images were retained. The number of adherent LO-EPC was counted and expressed as adherent cells per square millimetre. Video-microscopic recordings were made and analyzed offline using computerized image analysis software (Image ProPlus and Image J). The interactions of LO-EPC with HAEC were easily observed on the video and individual cell motion including rolling, tethering, transient adhesion, and firm arrest was recorded. The flow experiments were conducted at 37°C within a Perspex chamber.

### 2.3. Interaction of LO-EPC and ECM under* In Vitro* Shear Flow

100 *μ*g/*μ*L of collagen IV (BD, UK), collagen I (BD, UK), fibronectin (Sigma, UK), vitronectin (Invitrogen, UK), or laminin (Sigma, UK) was preplated to Ibidi *μ*-Slide VI 0.4 Luer slides for 1 hour at 37°C. The Ibidi slide was then connected to the flow system as described above. The slide was washed for 2 min with 1% BSA in DPBS. A total of 4 × 10^5^ LO-EPC in 1.5 mL were perfused at a shear stress of 0.7 dyn/cm^2^ for 4 min. An additional 2 min wash was applied to remove the nonadherent cells. Cells were subsequently fixed with 4% paraformaldehyde for 15 min at room temperature, washed twice with PBS, and stained with Hoechst dye (1 *μ*g/mL) for 30 min. The number of adherent LO-EPC was imaged, counted, and expressed as adherent cells per square millimetre.

### 2.4. Static Interaction of LO-EPC with ECM

100 *μ*g/*μ*L of collagen IV, collagen I, fibronectin, vitronectin, or laminin was preplated to Ibidi *μ*-Slide VI 0.4 Luer slides for 1 hour at 37°C before rinsing twice with DPBS. 2 × 10^4^ LO-EPC were seeded on each ECM-treated Ibidi slide and incubated at 37°C for 45 min. After washing twice with DPBS, cells were fixed with 4% paraformaldehyde for 15 minutes at room temperature and stained with Hoechst for 30 min. The total number of adherent cells was imaged, counted, and expressed as adherent cells per square millimetre.

### 2.5. Flow Cytometric Analysis of Cell Surface Markers

To study TNF*α* mediated HAEC activation, control HAEC and HAEC treated with 0.05 ng/*μ*L TNF*α* (R&D System, UK), for 4 hours at 37°C, were collected in 100 *μ*L 1% bovine serum albumin (BSA, Sigma) in PBS and incubated with fluorescein conjugated anti-human E-selectin antibodies (R&D System), APC conjugated anti-human ICAM-1 (BD), and PE/Cy5 conjugated anti-human VCAM-1 (Bio Legend, UK), together with the respective isotype control antibodies. Forward-side scatter plots were used to exclude dead cells. Data analysis was performed using CELL Quest software (BD) and FlowJo.

The expression of VE-Cadherin in LO-EPC and HAEC was quantified by flow cytometry using a FITC conjugated antibody against human VE-Cadherin (Abcam, UK), together with its corresponding isotype.

### 2.6. Immunofluorescence Staining

VE-Cadherin expression was visualized by immunofluorescence staining. Control HAEC and HAEC treated with ischaemia reperfusion injury were fixed for 15 min in 4% paraformaldehyde. Cells were then blocked for 30 min in 3% (w/v) BSA in TBS at pH 7.4 (blocking buffer). FITC conjugated VE-Cadherin antibodies (Abcam, UK) were diluted 1 : 50 in blocking buffer and incubated with cells at 4°C overnight.

### 2.7. Cell Staining of Dil-Acetylated-Low Density Lipoprotein (Dil-Ac-LDL)

Both endothelial cells and LO-EPC can take up Dil-Ac-LDL. To label LO-EPC with Dil-Ac-LDL, LO-EPC were incubated with 10 *μ*g/mL of Dil-Ac-LDL (Molecular Probes, Invitrogen) for 1 hour at 37°C and then washed twice with PBS. Labelled LO-EPC were distinguished from HAEC after adhesion.

### 2.8. VE-Cadherin Blocking Studies

Purified mouse antibody against human VE-Cadherin was used to block the surface expression of VE-Cadherin in HAEC (clone: BV9, Biolegend, UK). 3 × 10^4^ HAEC were incubated with 50 *μ*g/mL of antibody for 1 hour at 37°C before being connected to the flow system as described above. The cells were washed for 2 min with 1% BSA in DPBS prior to perfusion, with a total of 4 × 10^5^ LO-EPC in 1.5 mL of 1% BSA in DPBS.

### 2.9. Ischaemia Reperfusion Injury

Ischaemia reperfusion injury was simulated by anoxic (O_2_ < 1% and CO_2_ > 5%) and acidotic conditions with glucose and pyruvate deprivation as described previously [29]. LO-EPC were incubated with a minimal volume of ischaemia solution (118 mM NaCl, 24 mM NaHCO_3_, 1 mM NaH_2_PO_4_·H_2_O, 2.5 mM CaCl_2_·2H_2_O, 1.2 mM MgCl_2_, 0.5 mM sodium·EDTA·2H_2_O, 20 mM sodium lactate, and 16 mM KCl, pH 6.2) under hypoxia in an anaerobic bag (BDH), at 37°C for 4 h. Cells were then transferred to a 37°C incubator with 5% CO_2_ with additional complete culture medium for reperfusion overnight.

### 2.10. Integrin Blocking Studies

1.5 mL of 4 × 10^5^ LO-EPC was incubated with 10 *μ*g/mL anti-ITG *α*5*β*1 (Millipore, UK), anti-ITG *α*V*β*3 (Millipore, UK), and anti-ITG *α*v*β*1 (Bioss, Antibodies-online.com) or no antibodies (control) for 30 minutes at 37°C with gentle rotation. LO-EPC were then perfused onto fibronectin pretreated Ibidi slides under a shear stress of 0.7 dyn/cm^2^. After perfusion, the slides were washed for an additional 2 min to remove nonadherent cells. Adherent cells were then fixed with 4% paraformaldehyde and stained with Hoechst. The cells were imaged, counted, and expressed as adherent cells per square millimetre.

### 2.11. Live Cell Image Acquisition and Analysis

Live cell imaging of the interaction of LO-EPC with HAEC or ECM was performed using a digital imaging system coupled to an inverted microscope under flow conditions. The camera was set up to observe the top view of the rolling of LO-EPC on endothelial cells or ECM. The images were acquired though Image Pro software. The images were taken from a representative field of view every 30 seconds for 5 min from the start of LO-EPC perfusion. The sequence of events including LO-EPC rolling, tethering, and binding was recorded. The total number of adherent LO-EPC was stained with Hoechst or Dil-Ac-LDL and determined by counting the total adherent cells in 3–6 fields of view. The rolling velocity was observed but no specific measurements were recorded in this study.

### 2.12. Statistical Analysis

All values are expressed as mean ± SE from at least three separate experiments. Within each independent experiment, at least duplicate measurements were performed. One way ANOVA with Newman Keuls* post hoc* test was used to determine significance for all experiments. A probability value of *p* < 0.05 was considered statistically significant and is indicated by *∗*, and *p* < 0.01 is indicated by *∗∗*.

## 3. Results

### 3.1. Interaction of LO-EPC with Human Abdominal Aorta Endothelial Cells under Flow

**Figure 1:**
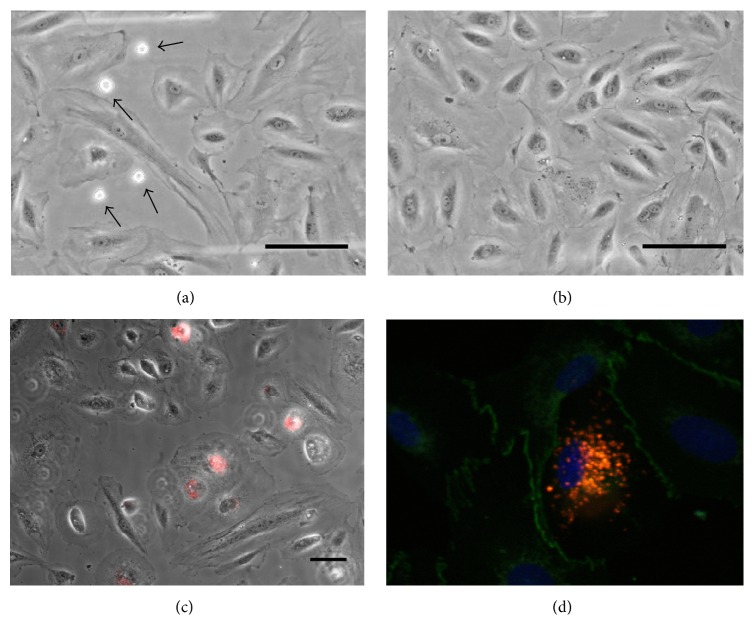
Representative phase-contrast live cell images of interaction of LO-EPC with 3 × 10^4^ subconfluent (a) and 6 × 10^4^ confluent (b) HAEC monolayers under shear stress 0.7 dyn/cm^2^. 4 × 10^5^ LO-EPC were perfused for 4 min. Arrows indicated adherent LO-EPC which only adhered paracellularly at junctional regions of discontinuity between two cells in subconfluent HAEC monolayer. Scale bar 30 *μ*m. After adhesion LO-EPC spread and establish cell-cell interaction (c). LO-EPC were labelled with DiI-Ac-LDL shown red. Scale bar 60 *μ*m. VE-Cadherin staining revealed the formation of lateral junction between LO-EPC and HAEC (d). LO-EPC were labelled with DiI-Ac-LDL shown red and VE-Cadherin expression shown green.

The interaction of LO-EPC with a human abdominal aortic endothelial cell (HAEC) monolayer was assessed under 0.7 dyn/cm^2^ shear stress at 37°C.  Figure 1(a) shows LO-EPC adhere to a subconfluent HAEC monolayer (3 × 10^4^) at gaps between adjacent HAEC paracellularly rather than adhering to superficial (luminal) surface of the HAEC. Interaction did not occur on a completely confluent HAEC monolayer (6 × 10^4^) (Figure 1(b)). The number of cells adhering to complete confluent and subconfluent HAEC was 3.25 ± 0.38 and 21.46 ± 1.81/mm^2^, respectively. Dilution of the seeded HAEC to 1 × 10^4^ to increase the intercellular spacing between HAEC decreased the adhesion of LO-EPC to HAEC (data not shown), suggesting that LO-EPC preferentially form adjacent contacts with HAEC. Similar rolling velocity was observed regardless of HAEC confluence. The adherent LO-EPC appeared as round cells initially, but they could withstand the shear forces, spread rapidly after firm adhesion, and start to establish cell-cell connections under 0.7 dyn/cm^2^ shear stress (Figure 1(c)). Immunofluorescence staining of VE-Cadherin in adherent LO-EPC revealed adhesion junction formation between LO-EPC and HAEC (Figure 1(d)), while Claudin-5 (tight junction protein) and PECAM showed more diffuse staining in adherent LO-EPC (data not shown), suggesting VE-Cadherin promotes a homotypic type of recognition between LO-EPC and HAEC. Adhesion of LO-EPC did not disrupt the HAEC morphology and monolayer structure and no transmigration of LO-EPC was observed. This was in contrast to monocytes, which interacted only by binding superficially to a confluent HAEC layer and then transmigrating (data not shown).

### 3.2. The Interaction of LO-EPC with HAEC Was Not Adhesion Molecule-Dependent under Flow

**Figure 2:**
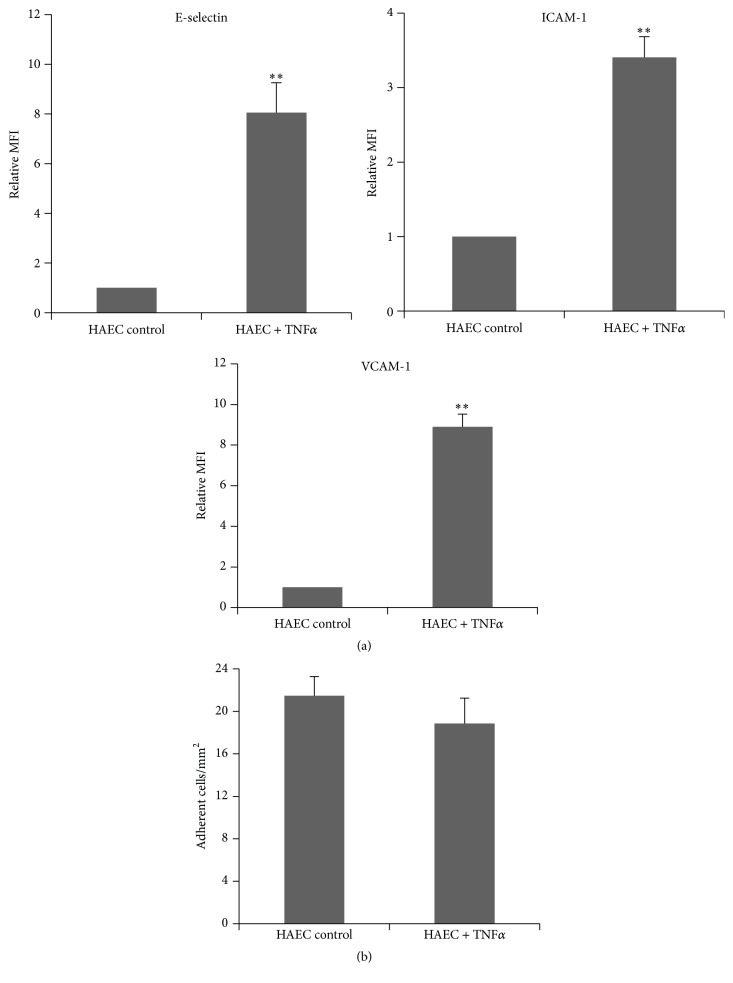
Interaction of LO-EPC with activated HAEC. Surface expression of adhesion molecules in HAEC after stimulation with 0.05 ng/mL TNF*α* (a). Surface expressions of E-selectin, ICAM-1, and VCAM-1 were quantified using flow cytometry. The graph shows relative Mean Fluorescence Intensity (MFI) normalised to untreated cells and represents the mean ± SE of three experiments. ^*∗∗*^ *p* < 0.01. Activation of HAEC did not increase the interaction with LO-EPC (b). 4 × 10^5^ LO-EPC were perfused to 3 × 10^4^ HAEC for 4 min and the data represented as mean ± SE of three experiments.

TNF*α* (0.05 ng/mL) was used to induce HAEC activation to investigate whether this enhanced the interaction with LO-EPC. HAEC activation was characterised by increased expression of cell surface adhesion molecules E-selectin, ICAM-1, and VCAM-1 in HAEC (Figure 2(a)). Activation did not increase the interaction with LO-EPC under 0.7 dyn/cm^2^ shear stress (Figure 2(b)) and there was no difference seen in rolling velocity, suggesting that adhesion molecules did not mediate LO-EPC rolling or adhesion to HAEC under flow conditions. This contrasted with monocytes in which the interaction increased when HAEC were activated. When 10^6^ monocytes were perfused the number of adherent monocytes increased from 6.01 ± 0.67 cells per millimetre square in untreated HAEC to 45.74 ± 4.03 cells per millimetre square in activated HAEC (*p* < 0.01) under 0.7 dyn/cm^2^ shear stress.

### 3.3. Vascular Endothelial- (VE-) Cadherin Mediates the Interaction of LO-EPC with HAEC under Flow

**Figure 3:**
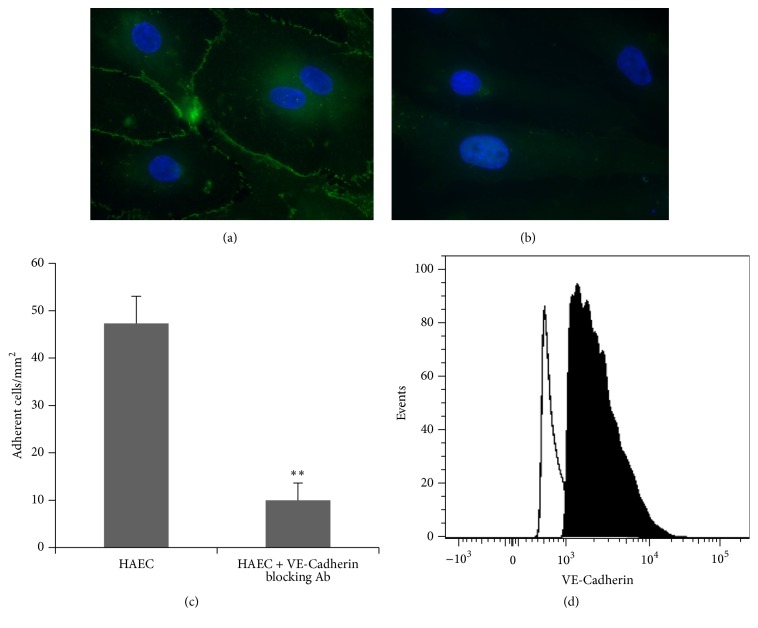
Representative microscopic images of VE-Cadherin expression in control HAEC (a) and HAEC incubated with anti-VE-Cadherin antibody for 1 hour at 37°C (b). Scale bar 20 *μ*m. Effect of VE-Cadherin on dynamic interaction of LO-EPC with HAEC. 4 × 10^5^ LO-EPC were perfused (c). The data was represented as mean ± SE of three experiments. ^*∗∗*^ *p* < 0.01. Representative flow cytometric histograms illustrating VE-Cadherin expression in LO-EPC and HAEC (d). The lined histogram represents VE-Cadherin expression in HAEC and the filled histogram represents VE-Cadherin expression in LO-EPC.

VE-Cadherin is an endothelium specific adhesion protein prominently located at junctions between endothelial cells suggesting it may play a role in initiating the interaction of LO-EPC with HAEC. We showed that LO-EPC had higher expression levels of VE-Cadherin compared to HAEC (Figure 3(d)). Higher expression of VE-Cadherin could contribute to LO-EPC adherence to HAEC paracellularly.

To investigate the involvement of VE-Cadherin in the interaction of LO-EPC with HAEC under dynamic flow, an antibody against VE-Cadherin was used to block the surface expression of VE-Cadherin (Figures 3(a) and 3(b)). Blocking VE-Cadherin in HAEC reduced the interaction of LO-EPC with HAEC significantly (Figure 3(c)), without significantly changing the rolling velocity (observation only), confirming the role of VE-Cadherin in LO-EPC adhesion to HAEC under 0.7 dyn/cm^2^ shear stress.

### 3.4. Decreased Interaction of LO-EPC with HAEC after Ischaemia Reperfusion Injury under Flow

**Figure 4:**
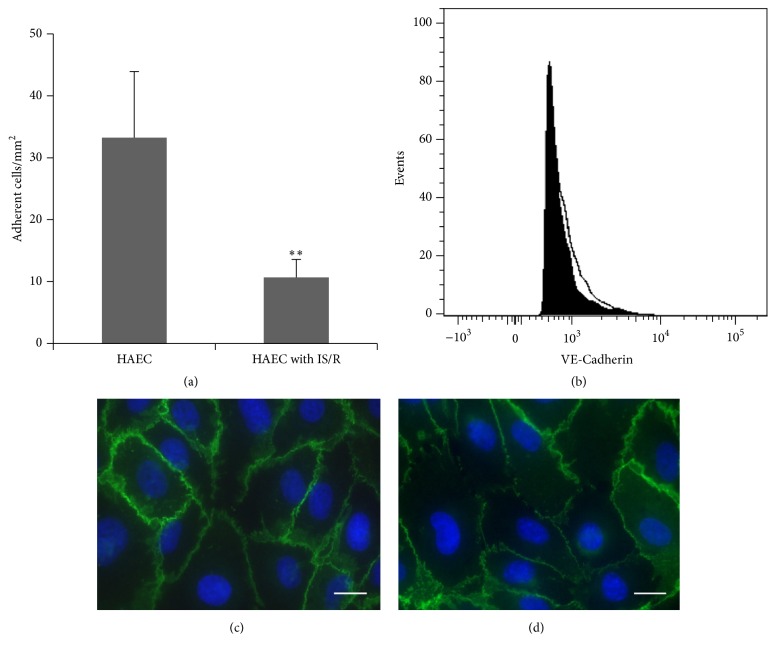
Dynamic interaction of LO-EPC with control HAEC and HAEC with ischaemia reperfusion injury (a). The data represented as mean ± SE of three experiments. ^*∗∗*^ *p* < 0.01. Representative flow cytometric histograms of VE-Cadherin expression in control HAEC and HAEC with ischaemia reperfusion injury (b). The filled histogram represents VE-Cadherin expression in control HAEC and the unfilled histogram represents VE-Cadherin in HAEC with ischaemia reperfusion injury. Representative microscopic images of VE-Cadherin expression in control HAEC (c) and HAEC with ischaemia reperfusion injury (d). Scale bar 20 *μ*m.

HAEC treated with simulated ischaemia reperfusion* in vitro* under flow showed decreased interaction with LO-EPC compared to normal HAEC (Figure 4(a)). Four hours of ischaemia followed by reperfusion caused HAEC retraction and detachment, also demonstrated by significantly more floating cells in the supernatant compared to untreated control HAEC. There was no significant difference in VE-Cadherin expression between control HAEC and HAEC with ischaemia reperfusion injury (Figures 4(b)–4(d)). This suggests that the decreased interaction between LO-EPC and HAEC after ischaemia reperfusion injury may be due to cell retraction and increased intercellular space between HAEC rather than being VE-Cadherin related.

### 3.5. Interaction of LO-EPC with Extracellular Matrix Proteins

**Figure 5:**
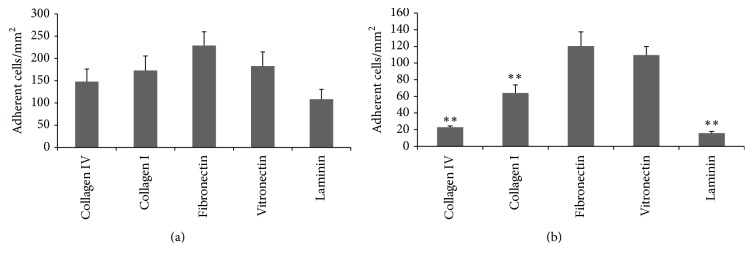
LO-EPC adhesion to various extracellular matrix proteins under static and flow conditions. 2 × 10^4^ of LO-EPC were plated to Ibidi *μ*-Slide VI 0.4 Luer slides for 45 min to study the static adhesion of LO-EPC to various ECM (a). Dynamic adhesion of LO-EPC to various extracellular matrix proteins under 0.7 dyn/cm^2^ shear stress (b). 4 × 10^5^ EPC were perfused into Ibidi *μ*-Slide VI 0.4 Luer slides coated with 100 *μ*g/mL collagen IV, collagen I, fibronectin, vitronectin, and laminin, respectively. The data was represented as mean ± SE of three experiments. ^*∗∗*^ *p* < 0.01 compared to fibronectin.

When injured endothelial cells retract and/or detach, interstitial basal membrane is exposed. Endothelial basal membranes are comprised of several extracellular matrix (ECM) proteins, including collagen IV, collagen I, fibronectin, vitronectin, and laminin. We compared the adhesion of LO-EPC to different ECM. There was no significant difference in LO-EPC binding to collagen IV, collagen I, fibronectin, and vitronectin under static conditions (Figure 5(a)). Under 0.7 dyn/cm^2^ shear stress, however, LO-EPC bind to different ECM with different strengths, with a higher adhesive strength for fibronectin and vitronectin (Figure 5(b)). There were no signs of toxicity of these substrates on LO-EPC. In addition LO-EPC adhere more avidly to fibronectin and vitronectin than they do to HAEC (Figure 4(a)).

Differences in rolling velocity were observed when examining the motion of LO-EPC interaction with ECM under the microscope. The rolling of LO-EPC on fibronectin and vitronectin coated surfaces was slower (data not shown), suggesting that fibronectin and vitronectin influenced both rolling and adhesion phases of interaction with LO-EPC.

**Figure 6:**
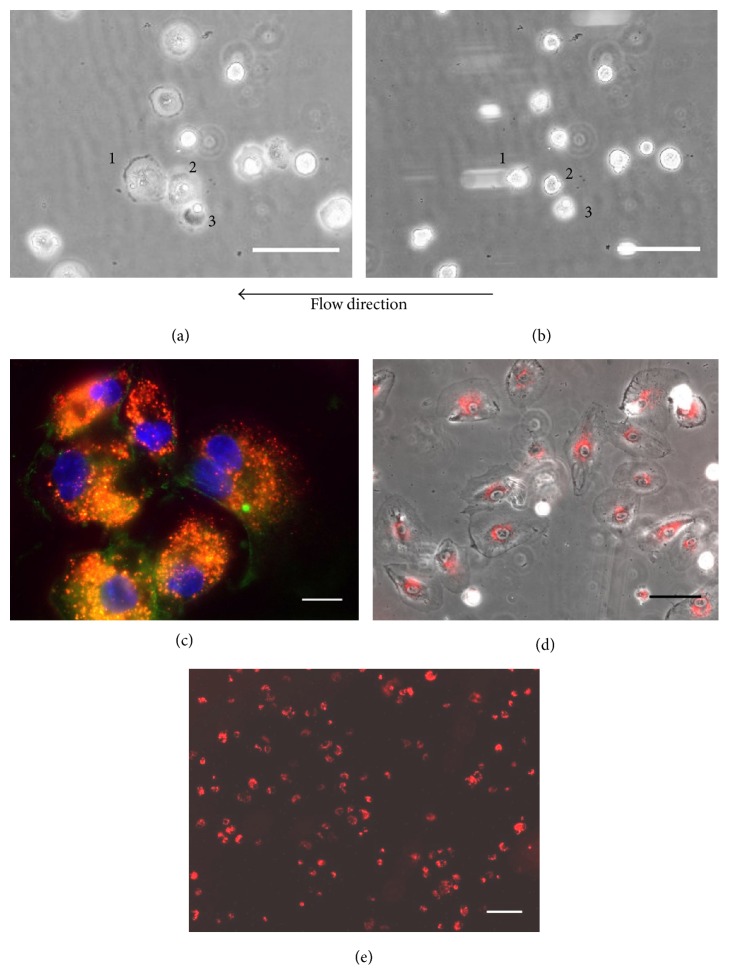
LO-EPC (4 × 10^5^) were perfused over fibronectin-coated Ibidi slides and time lapse imaging was used to visualise and record LO-EPC adhesion. Representative still images show three adherent cells after initial capture (a, b) after 4 min of perfusion (0.7 dyn/cm^2^). Scale bar represents 30 *μ*m. Adherent LO-EPC form lateral adhesion junction (c). LO-EPC labelled with DiI-Ac-LDL shown red and VE-Cadherin expression shown green. Scale bar 20 *μ*m. 24 hours after adhesion to fibronectin, LO-EPC spread and proliferate (d). Scale bar represents 60 *μ*m. The adherent LO-EPC effectively cover a fibronectin-coated surface (e). Scale bar represents 110 *μ*m.

Figure 6(a) shows that, under a shear stress of 0.7 dyn/cm^2^, LO-EPC readily attach and spread on a fibronectin coated surface. Rolling LO-EPC appeared as round cells initially, but rapidly spread, formed cell-cell connections upon firm adhesion, and withstood a total of 10 min perfusion under flow (Figures 6(a) and 6(b)). LO-EPC displayed coordinated adhesion behaviour under flow with sequential events of rolling along the surface for short distances and episodes of transient tethering prior to firm adherence. Immunofluorescence staining of VE-Cadherin in adherent LO-EPC confirmed the formation of lateral junctions (Figure 6(c)). 24 hours after adhesion, LO-EPC had proliferated (Figure 6(d)) and a large surface area was covered by adherent LO-EPC (Figure 6(e)).

### 3.6. Exposed Fibronectin Enhanced the Cell-Cell Interaction between LO-EPC and HAEC

**Figure 7:**
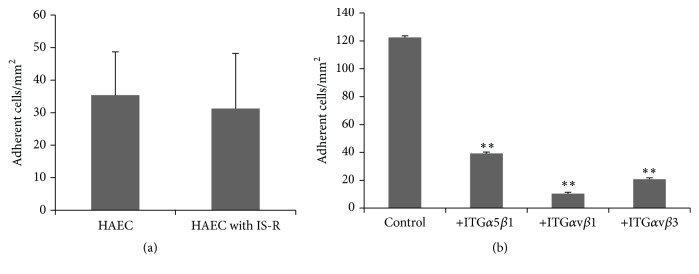
The influence of fibronectin on the dynamic interaction of LO-EPC with control HAEC and HAEC with ischaemia reperfusion injury under 0.7 dyn/cm^2^ shear stress. 3 × 10^4^ HAEC were plated into Ibidi *μ*-Slide VI 0.4 Luer slides which had been precoated with 100 *μ*g/mL fibronectin. The data represent a mean ± SE of three experiments. LO-EPC adhesion to fibronectin was integrin dependent (b). LO-EPC binding to fibronectin was blocked by antibodies against integrin *α*5*β*1, integrin *α*v*β*1, and integrin *α*v*β*3. The data represent the mean ± SE of three experiments. ^*∗∗*^ *p* < 0.01 compared to the control (no blocking antibodies).

Interaction of LO-EPC with HAEC that had undergone ischaemia reperfusion was increased when HAEC had been seeded on to fibronectin (Figures 7(a) and 4(a)). There was no significant difference between the dynamic interactions of LO-EPC with control HAEC and HAEC with ischaemia reperfusion injury (Figure 7(a)), suggesting fibronectin could promote the cell-cell interaction of LO-EPC with a retracted or discontinuous HAEC in order to aid in the reformation of the endothelial cell monolayer.

### 3.7. Integrins Mediate Adhesion of LO-EPC on Extracellular Matrix Proteins under Flow

We have shown previously that there is differential integrin gene expression in LO-EPC, with higher expression of integrin monomers *α*v, *α*5, *β*1, and *β*3 and higher cell surface expression of integrin heterodimers *α*5*β*3, *α*v*β*1, and *α*v*β*3 [16]. Using blocking antibodies against integrins *α*5*β*3, *α*v*β*1, and *α*v*β*3, the interaction of LO-EPC with fibronectin was significantly decreased. The data suggested that the interaction between LO-EPC and ECM was mediated largely by these three integrins *α*5*β*3, *α*v*β*1, and *α*v*β*3 (Figure 7(b)).

## 4. Discussion

Adhesion of LO-EPC to injury sites involves both cell-cell and cell-matrix interactions. Enabling direct interaction between endothelial cells and ECM is likely critical for LO-EPC homing and performing vascular repair at the injury site. The most extensively studied cell-cell interaction under dynamic flow is the interaction of leukocytes with endothelial cells [27, 30, 31]. Studies on the dynamic interaction of early outgrowth EPC with endothelial cells showed a strong resemblance to that of leukocyte interactions with activated endothelial cells; they share some common features of a coordinated sequence of multistep adhesive events including an initial phase of rolling and final firm adhesion [32, 33]. The initial phase of leukocyte rolling* in vivo* is mediated by P-selectin and firm adhesion is mediated by E-selectin, ICAM-1, and VCAM-1 [32, 33]. Until now there has been no information on the dynamic interaction of LO-EPC with endothelial cells. Using an* in vitro* flow system to simulate physical conditions of blood circulation* in vivo*, we showed that LO-EPC did not interact with confluent EC under flow but readily adhered and spread where there were discontinuities in the EC monolayer. The interaction occurred paracellularly at gaps in the intercellular junctions between EC and was not critically adhesion molecule-dependent since upregulating the cell surface adhesion molecules E-selectin, ICAM-1, and VCAM-1 in HAEC did not alter the interaction of LO-EPC with EC under flow. The adhesion mechanism is distinct and in contrast to the interaction of early outgrowth EPC [33], monocytes [31], and mesenchymal stem cells (MSC) [34] in which the interactions with endothelial cell were all adhesion molecule-dependent. One possible explanation for the apparent different adhesion mechanism used by LO-EPC may be the integrin expression profile of LO-EPC. LO-EPC show low expression of integrins *α*L*β*2, *α*M*β*2, *α*X*β*2, and *α*D*β*2 [16], which are responsible for binding to vascular ligands such as ICAM-1, ICAM-2, and VCAM-1 [35, 36]. LO-EPC also have lower expression of integrin *α*4*β*1, whereas MSC engage VLA-4 (integrin *α*4*β*1)/VCAM-1 to mediate firm adhesion on EC [34]. Different cells may use different adhesion mechanisms depending on the respective adherent properties as demonstrated previously in the interaction of tumour cells and endothelial cells. Cells from different tumour types interact with the endothelial surface using different mechanisms depending on adhesion molecules expressed on the tumour and endothelial cell surface [37].

The interaction of LO-EPC with HAEC occurred paracellularly suggesting that cell-cell contact on lateral surfaces may play a role in initiating this interaction. Vascular endothelial- (VE-) Cadherin is a strictly endothelial specific adhesion junction protein, prominently localised at endothelial cell lateral borders and mediates homotypic cell-cell adhesion [38–40]. “Homophilic interactions” between LO-EPC and EC suggest that VE-Cadherin may mediate initial adhesion of LO-EPC to endothelial cells. VE-Cadherin, CD31, and CD146 are typically associated with a more mature endothelial phenotype; however we showed that LO-EPC had a higher level of VE-Cadherin compared to mature endothelial cells (HAEC), which may be significant in ensuring that LO-EPC resist tractive flow forces. The adhesion junction formation observed in the adherent LO-EPC with HAEC confirmed that the adhesion of LO-EPC with HAEC was at least partly mediated by VE-Cadherin. Interactions between VE-Cadherin activate the cellular cascade signalling pathways further strengthening the cadherin interaction [41]. Indeed, blocking VE-Cadherin in the endothelial cells reduced their interaction with LO-EPC interaction under dynamic flow. In addition, VE-Cadherin regulates various cellular processes such as cell proliferation and modulates vascular endothelial growth factor receptor functions as well as being involved in VE-Cadherin-mediated contact inhibition of cell growth [42, 43]; therefore LO-EPC would fulfil nearly every function required by reparative cells. A similar interaction pattern was also observed previously in the interaction of blood-borne tumour cells with endothelial cells. The preferred tumour cells interactions prior to tumour cell extravasation occur at sites near to endothelial intercellular junctions [44]. Although VE-Cadherin is a strictly EC specific adhesion molecule it is also expressed by aggressive melanoma tumours [45]. Development of long-term firm adhesions depends on the collaborative interactions of several adhesion proteins including tight junction protein and PECAM. Ayalon et al. showed that there were spatial and temporal relationships between VE-Cadherin and PECAM-1 in regulating endothelial cell-cell interaction [46]. Cadherins became organized on the cell surface much earlier than PECAM-1 and served as the nucleation sites for subsequent and adjacent assembly of PECAM-1 adhesions [46]. The reciprocal role of these junctional proteins in regulating stable junction organization and biological activity in the adherent LO-EPC remains to be clarified.

When endothelial cells are subjected to ischaemia reperfusion injury, the dynamic interaction of LO-EPC with EC was decreased. Koto et al. reported that hypoxia could disrupt the barrier function of neural blood vessels through changes in the expression of adhesion junction protein claudin-5 in endothelial cells [47]. However, our data showed that ischaemia reperfusion injury did not significantly influence VE-Cadherin expression in EC, suggesting that decreased interaction was unlikely to be due to disrupted VE-Cadherin function. This was in agreement with Chen et al. who showed that 4 hours of ischaemia did not cause significant changes in mRNA expression of VE-cadherin and claudin-5 in endothelial cells in the lung [48]. The decreased interaction we observed was likely due to an increase in the size and number of intercellular spaces caused by cellular retraction under these conditions. This is consistent with our observation that increasing the cell spacing of a monolayer also decreased LO-EPC interaction.

The extracellular matrix (ECM) beneath the endothelium is a highly organized complex network of collagens, fibronectin, vitronectin, laminin as well as proteoglycans, glycoproteins, and bound growth factors. They form a thin sheet-like matrix to create varying degrees of tissue tensile strength to preserve the function and integrity of blood vessels [22, 49–51]. When endothelial cells are damaged, ECM components will be exposed on the luminal surface. So far there has been little study of adhesion of LO-EPC to ECM under dynamic flow. Angelos et al. showed that LO-EPC could interact with fibronectin and the number of LO-EPC adhering to a fibronectin coated surface was influenced by the perfused cell density and shear stress [52]. In this study, we compared LO-EPC adhesion strength to different ECM and demonstrated that LO-EPC are highly adhesive to fibronectin and vitronectin but less so on collagen IV, collagen I, and laminin under a shear stress of 0.7 dyn/cm^2^. Different ECM interact with cells via different cell surface integrin receptors [53, 54]. We showed that the interaction of LO-EPC with fibronectin was strongly dependent on integrins *α*5*β*1, *α*V*β*1, and *α*V*β*3. The involvement of integrin *α*5*β*1 in LO-EPC with matrix protein is in agreement with other published works [22, 52] in which increased adhesion of LO-EPC to fibronectin was generated by LO-EPC producing multiple contacts of *α*5*β*1 with a fibronectin-coated surface and the contact area growing during the first 20 minutes of attachment [52]. Previously we showed that there was higher gene expression of integrin subunits *α*5, *α*v, *β*1, and *β*3, moderate expression of *α*6 and *α*E, and low level expression of other integrin subunits, and that integrins *α*5*β*1, *α*V*β*3, and *α*V*β*3 have higher cell surface expression in LO-EPC [16], the receptors for fibronectin (integrins *α*5*β*1, *α*V*β*1, and *α*V*β*3) [55] and vitronectin (integrin *α*V*β*3) [56]. The receptor for collagen IV and collagen I (integrins *α*1*β*1 and *α*2*β*1) [57, 58] and laminin (integrins *α*3*β*1, *α*6*β*1, *α*7*β*1, and *α*6*β*4) [58] were expressed at lower levels in LO-EPC. The constitution of endothelial basal membranes varies between different vascular beds and fibronectin and vitronectin are not normally involved in maintaining tissue structure and are found at lower levels in quiescent vessels [59, 60]. Both fibronectin and vitronectin are present in the bloodstream [61] with serum concentrations of 300 *μ*g/mL and 200–300 *μ*g/mL, respectively [62, 63]. When an endothelial cell monolayer is damaged, the cells leak into the injured area and are rapidly deposited in injured tissue, becoming a prominent constituent of the endothelial basement membrane, and provide an adhesive scaffold for the recruitment of cells [49, 62, 63]. The higher local concentrations of fibronectin and vitronectin after injury and the higher binding strength with LO-EPC make the injury site a strong target for capturing LO-EPC homing to an injury site.

Although in this study we examined the interaction of LO-EPC with HAEC and ECM separately, these two processes are closely linked and occur concomitantly, especially in the initial phase of vascular injury. Endothelial cells and the supporting matrix exist in a state of “dynamic reciprocity” to serve and regulate each other. ECM not only provides a substrate for cell attachment and spreading, contact guidance for cell migration, and a scaffold for building tissues but also serves as a reservoir for growth factors [64]. EC are primarily responsible for the synthesis and deposition of these ECM [64]. We found that fibronectin and vitronectin provided superior adhesion for LO-EPC compared to HAEC. Adhesion to ECM helped LO-EPC to establish junctional adhesion with HAEC as shown by EC with ischaemia reperfusion injury causing decreased dynamic interaction with LO-EPC which was restored when EC were seeded on fibronectin.

It was reported that mature endothelial cells increase deposition of collagen IV, fibronectin, and laminin under hypoxic condition which may contribute to the complex interplay between endothelial cells and ECM [50]. EPC deposited collagen IV, fibronectin, and laminin to a greater extent than mature EC [65]. Therefore, using autologous LO-EPC therapeutically could amplify these benefits and enhance endogenous repair.

*Limitations of This Study*. (1) In this study 0.7 dyn/cm^2^ shear stress was used to investigate the dynamic interaction of LO-EPC with endothelial cells and ECM. Angelos et al. showed that the number of adherent LO-EPC/cm^2^ exhibited a biphasic response with the optimal shear stress for late outgrowth EPC binding to fibronectin at 1 dyn/cm^2^ [52], a biphasic response similar to both neutrophils and monocytes binding to the endothelium under flow [66, 67]. The adhesive strength under flow not only depends on adhesive signals, but also depends on shear stress. Higher shear stress could interfere with the binding strength by increased rolling velocity or might help with binding if modelling of microvillus deformation is accurate [68]. Future studies will investigate the influence of different shear stresses on interaction of LO-EPC with endothelial cells and ECM.

(2) The model used in this study was a simplified one.* In vivo*, exposed endothelial basal membrane is not only a target for LO-EPC; but it will also attract platelets and other immune cells. Platelets aggregate immediately after endothelial denudation and adhere to ECM by platelet-specific integrin *α*IIb*β*3 [60]. Activated platelets play a role not only in thrombosis but also in inflammation, immune responses, and atherosclerotic disease [69]. Recently it was reported that activated platelets could also support adhesion and migration of circulating progenitor cells [70]. Platelet-coated ECM may represent an attractive adhesive surface promoting arrest of circulating CD34+ progenitor cells* in vitro* as well as* in vivo* [71]. Whether platelets and other inflammatory cytokines encourage or prevent LO-EPC interaction with endothelial cells and ECM under flow perfusion will be investigated in future experiments.

## 5. Conclusion

In conclusion, we have demonstrated that discontinuous endothelial monolayer and exposed ECM were sufficient adhesive signals to capture LO-EPC from flow perfusion* in vitro* and that LO-EPC demonstrate appropriate properties to effect vascular repair. Further studies are needed to examine whether these adhesive signals are effective under different shear stresses and strong enough to capture LO-EPC from blood circulation* in vivo*.
